# A Narrative Review of Neuroimaging Studies in Acupuncture for Migraine

**DOI:** 10.1155/2021/9460695

**Published:** 2021-11-10

**Authors:** Peihong Ma, Xiaohui Dong, Yuzhu Qu, Zhaoxuan He, Tao Yin, Shirui Cheng, Zilei Tian, Yuke Teng, Kunnan Xie, Ruirui Sun, Siyi Yu, Fang Zeng

**Affiliations:** ^1^Acupuncture and Tuina School/Acupuncture & Brain Science Research Center, Chengdu University of Traditional Chinese Medicine, Chengdu, Sichuan, China; ^2^Acupuncture and Moxibustion Department, Beijing University of Chinese Medicine, Beijing, China

## Abstract

Acupuncture has been widely used as an alternative and complementary therapy for migraine. With the development of neuroimaging techniques, the central mechanism of acupuncture for migraine has gained increasing attention. This review aimed to analyze the study design and main findings of neuroimaging studies of acupuncture for migraine to provide the reference for future research. The original studies were collected and screened in three English databases (PubMed, Embase, and Cochrane Library) and four Chinese databases (Chinese National Knowledge Infrastructure, Chinese Biomedical Literature database, the Chongqing VIP database, and Wanfang database). As a result, a total of 28 articles were included. Functional magnetic resonance imaging was the most used neuroimaging technique to explore the cerebral activities of acupuncture for migraine. This review manifested that acupuncture could elicit cerebral responses on patients with migraine, different from sham acupuncture. The results indicated that the pain systems, including the medial pain pathway, lateral pain pathway, and descending pain modulatory system, participated in the modulation of the cerebral activities of migraine by acupuncture.

## 1. Introduction

Migraine is a chronic neurological disease characterized by recurrent headaches and neurological symptoms [1]. As the third most prevalent disorder and the seventh highest specific cause of disability worldwide [2, 3], migraine not only affects the quality of life (QoL) and productivity of patients [4] but also results in a high financial burden to society [5]. Recently, acupuncture has been widely used as an alternative or complementary treatment for migraine with its efficacy and safety. Using the neuroimaging techniques to explore the underlying mechanism of acupuncture for migraine has also attracted the increasing interest of investigators.

Since the first neuroimaging study about acupuncture for migraine was published in 2008, nearly 30 studies have been emerged to improve the understanding of acupuncture for migraine with real-time visualized evidence. However, the designs and results of these studies showed significant differences. For example, some research studies concentrated on study designs from different perspectives, such as different manipulation modalities of acupuncture and different subtypes of migraine; several studies applied different neuroimaging scanning techniques or used different analytical methods. The various study designs may partly contribute to different results which affect the clinical application to some degree. Therefore, this review aimed to analyze the study design and summarize the main results of the published neuroimaging studies in acupuncture for migraine to deepen the understanding of the cerebral mechanism of acupuncture for migraine and provide reference and guidance for future research.

## 2. Methods

### 2.1. Searching Strategy

Data collections were conducted in three English language databases (PubMed, Embase, and Cochrane Library) and four Chinese language databases (Chinese National Knowledge Infrastructure, CNKI; Chinese Biomedical Literature database, CBM; the Chongqing VIP database, VIP; and the Wanfang database, WF) from database inception up to 31st Dec 2020, with the combination of the main keywords: neuroimaging, acupuncture, and pain; studies about migraine were selected. The retrospective searching was performed on reference lists of included articles. Details of search terms were depicted and modified for each database in Supplementary Table 1.

The article was included if [1] the studies were original articles, [2] the patients were diagnosed with migraine, [3] acupuncture was the intervention method, and [4] neuroimaging techniques were used to investigate the cerebral changes elicited by acupuncture (Supplementary Table 1).

The article was excluded if [1] it was a duplicate article or [2] it was the irrelevant article, review, case report, protocol, only abstract, editorial letter, retracted article, and animal experiment. Studies not fulfilling each of the above criteria were excluded (Supplementary Table 1).

Supplementary Figure 1 shows the flow diagram of the literature search and screening process.

### 2.2. Data Extraction and Analysis

The published year, the institution of corresponding authors (Supplementary Table 2 and Supplementary Figure 2), study design (types of migraine, interventions (manipulation modality and course of treatments), types of control, and clinical scales of migraine) (Supplementary Table 3), and the related neuroimaging information (neuroimaging scanning techniques, analytical methods of neuroimaging data, and the results of brain regions involved in acupuncture analgesia for migraine) (Supplementary Table 4) were extracted.

## 3. Results

Twenty-eight original articles were included in this review.

### 3.1. The Basic Information of the Studies

The first acupuncture-neuroimaging study for migraine was published in 2008. All the twenty-eight studies were published by Chinese researchers. The top three corresponding affiliations were Chengdu University of TCM (11 studies) [6–16], Dongzhimen Hospital of Beijing University of TCM (5 studies) [17–21], and Xidian University (3 studies) [22–24].

### 3.2. The Subtypes of Migraine Patients

**Figure 1:**
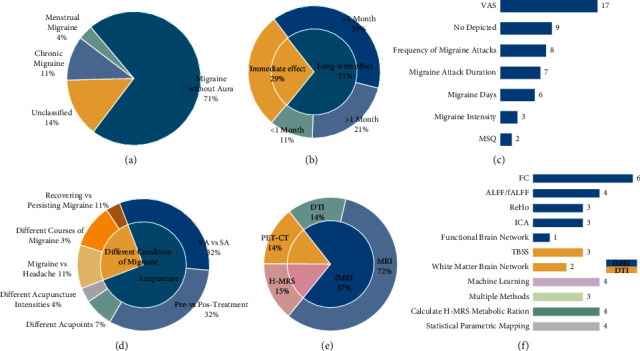
The study design of included studies. (a) The proportion of different types of migraine. (b) The proportion of treatment session. (c) The proportion of clinical variables of migraine. (d) The proportion of control types. (e) The proportion of scanning techniques. (f) The proportion of analysis methods of neuroimaging data. VA, verum acupuncture; SA, sham acupuncture; VAS, visual analogue scale; MSQ, Migraine-Specific Quality of Life Questionnaire; H-MRS, proton magnetic resonance spectroscopy; PET-CT, positron emission tomography-computed tomography; MRI, magnetic resonance imaging; fMRI, functional magnetic resonance imaging; DTI, diffusion tensor imaging; ALFF, amplitude of low-frequency fluctuations; FC, functional connectivity; ReHo, regional homogeneity; ICA, independent components analysis; TBSS. tract-based spatial statistics.

Twenty studies (71%) enrolled migraine patients without aura [6–8, 11, 14–29]. Four studies (14%) did not describe the subtypes of migraine [12, 30–32], three studies (11%) enrolled chronic migraine patients [9, 10, 33], and one study (4%) enrolled the patients with menstrual migraine [13] (Figure1(a)).

### 3.3. The Acupuncture Intervention

Twenty studies (71%) focused on the long-term efficacy of acupuncture for migraine [6–8, 11, 13, 15–17, 20, 22–25, 27–33]; eight studies (29%) adopted performed treatment session to explore the immediate efficacy of acupuncture for migraine [9, 10, 12, 14, 18, 19, 21, 26] (Figure1(b)).

Twenty-one (75%) studies were designed to concentrate on the central mechanisms of acupuncture for migraine. Among them, nine studies (32%) were designed as the self-control model (pre vs. posttreatment) [9, 10, 15, 18–21, 28, 29], nine studies (32%) explored the differences in cerebral responses between verum acupuncture (VA) and sham acupuncture (SA) [6–8, 12, 13, 16, 26, 27, 33], and the remaining three studies for different acupuncture intensities or different acupoints. Other studies concentrated on the central mechanisms of acupuncture for different conditions of migraine (Figure1(d)).

Seventeen studies used the visual analogue scale (VAS) to assess the pain intensity of migraine [6–8, 11–15, 17, 20, 22–24, 27–29, 33]. Besides, the condition of migraine such as frequency of migraine attacks (8 studies) [6–8, 11, 13, 16, 20, 29], migraine attack duration (7 studies) [20, 22–24, 28, 30, 31], migraine days (6 studies) [11, 15, 22–24, 29], and migraine intensity (3 studies) [13, 30, 31] was also assessed. Two studies adopted the disease-specific scale, Migraine-Specific Quality of Life Questionnaire (MSQ), to evaluate the QoL of migraine [22, 25] (Figure1(c)).

### 3.4. Neuroimaging Scan and Data Analysis

Twenty studies selected magnetic resonance imaging (MRI) to explore the cerebral responses of acupuncture for migraine [ 6–8, 11, 13, 15–29]; among these studies, fifteen studies applied functional magnetic resonance imaging (fMRI) [6–8, 11, 13, 15, 16, 18–21, 26–29], five studies applied diffusion tensor imaging (DTI) [17, 22–25], four studies used positron emission tomography-computed tomography (PET-CT), and four studies selected proton magnetic resonance spectroscopy (H-MRS) to investigate the metabolic ration of H-MRS to explore the cerebral responses of acupuncture for migraine (Figures 1(e) and 1(f)) [30–33].

Functional integration analysis (functional connectivity, independent component analysis, and functional brain network) (9 studies) were the common analytical methods applied in the fMRI [7, 8, 13, 16, 19, 20, 24, 28, 29] and followed by functional segregation analysis (amplitude of low-frequency fluctuations/fractional amplitude of low-frequency fluctuations, ALFF/fALFF; regional homogeneity, ReHo) (7 studies) [6, 11, 15, 18, 21, 26, 27]. Tract-based spatial statistics (TBSS) was the common analytical method applied in the DTI. Besides, the statistical parametric mapping (SPM) software was applied to assess the brain glucose metabolism alterations of acupuncture for migraine [9, 10, 12, 14]. Furthermore, three studies used multiple analytical methods [8, 22, 24] and four studies selected machine learning [15, 16, 22, 24].

**Figure 2:**
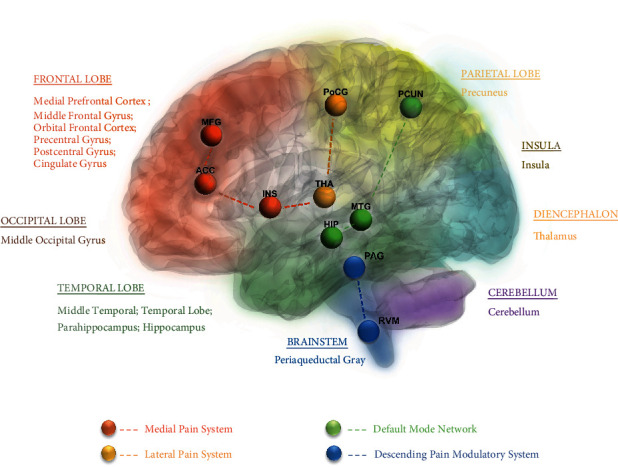
The main findings of acupuncture for migraine by neuroimaging techniques. The different shadow colors represent the different functional classifications of the brain. The high frequent reported areas that have been affected by acupuncture for migraine in the included studies were noted with different colors. The different color nodes represent the different pathways or networks. ACC, anterior cingulate cortex; DMN, default mode network; HIP, hippocampus; INS, insula; MFG, middle frontal gyrus; MTG, medial temporal gyrus; PAG, periaqueductal gray; PCUN, precuneus; PoCG, postcentral gyrus; RVM, rostral ventromedial medulla.

The cerebral responses to acupuncture for migraine are given in Supplementary Table 4. The high-frequency reported brain regions are shown in Figure 2.

## 4. Discussion

Since neuroimaging techniques were used to explore the mechanisms of acupuncture, the central mechanism of acupuncture for migraine has gradually drawn the attention of investigators. Since the first study explored the central mechanism of acupuncture for migraine, 28 studies have been published during the past 15 years. From the beginning, these articles simply concentrated on the self-control design of the cerebral activities, gradually developed to explore the different cerebral activities of the acupuncture efficacy (such as VA and SA), and now paid attention to the different cerebral activities of acupuncture for subtypes or stages of the migraine. These articles significantly improved the understanding of acupuncture for migraine with real-time visualized evidence.

### 4.1. The Study Design of Acupuncture-Neuroimaging Studies for Migraine

The design of the acupuncture-neuroimaging study for migraine could be divided into four categories according to the contents.

First, at the early stage, these studies [8–10, 18–21, 28, 29] mainly designed the self-controlled trial to investigate whether acupuncture could elicit cerebral responses on patients with migraine, accounting for more than one-third of studies. Although these studies only set one group to observe the cerebral signal changes comparing the brain activities at the baseline with that at the end of acupuncture therapy, these studies provided preliminary references for future studies. However, this kind of design had limitations what factors (such as the efficacy of acupuncture and moxibustion, placebo effect of acupuncture, or the natural course of migraine) caused the alterations of cerebral activities were still unclear.

Second, people gradually tended to investigate whether the cerebral changes elicited by acupuncture were different from those by SA, to explore the different cerebral activities between the acupuncture efficacy and placebo efficacy. The majority of these studies selected functional connectivity (FC) to analyze the neuroimaging data. FC has developed to the popular direction of the neuroimaging analytical method in recent years. However, few kinds of research concentrated on the depth, the intensities of acupuncture stimulation, or other factors that influenced the efficacy of acupuncture for migraine.The application of FC based on the specific ROI of the brain, rather than the whole brain, is a substantial limitation of these studies. Thus, it is valuable to explore the central mechanism of acupuncture for migraine from different perspectives including the influencing factors and large-scale whole brain networks.

Third, some studies investigated the central mechanism of acupuncture therapy for some subtypes (menstrual migraine or some stages of migraine). That means investigators mostly focused on the central mechanisms of acupuncture for specific subtypes of migraine. It has been proved that the cerebral activities of the ictal or interictal phase of the migraine existed differently [34–36]. The migraine with aura, chronic migraine, different phases of migraine was hardly explored. Therefore, future studies can explore other subtypes of migraine and apply the experimental pain to model the different phases of migraine to fully explore the cerebral responses to acupuncture for migraine.

Fourth, with the application of the machine learning (ML) algorithm in neuroimaging studies, people gradually began to predict the efficacy of acupuncture for migraine with neuroimaging data by the multivariate pattern analysis method. In this review, four studies applied ML to explore the structural and functional properties of the brain whether it can contribute significant information to predict the efficacy of acupuncture for migraine at the individual level [15, 16, 22, 24]. Unlike the univariate group-level statistics, ML can predict the efficacy of acupuncture for migraine with valid multivariate characteristics and provide specific clinical guidance at the individual level, which has gradually attracted increasing attention by researchers in recent years. Therefore, integrating ML and neuroimaging techniques will be the hot direction to identify neural “biomarkers” from neuroimaging data automatically at the individual level and predict the acupuncture efficacy for migraine.

Of the included studies, VAS was the most used scale for pain assessment for migraine. Besides, the clinical efficacy of migraine mostly focused on symptoms such as the frequency, intensity, and attack duration of migraine. Only five studies applied emotional scales and two studies applied disease-specific scales, MSQ, to assess the emotion and QoL of migraine patients. The pain experience is multifaceted and complex, encompassing multiple dimensions including psychological, cognitive, perception, affective, and behavior [37]. Except for the pain, the impaired functioning, psychological responses, and participant ratings of overall improvement are also associated with migraine [38–40]. Therefore, the multidimensional characteristics of migraine such as psychological, physical functioning, and participant ratings of overall improvement should be assessed simultaneously to explore the central mechanisms of the analgesic efficacy of acupuncture for migraine.

### 4.2. The Neuroimaging Methods Applied in the Acupuncture for Migraine

In this review, the neuroimaging techniques including MRI, PET, and H-MRS were applied. fMRI was the most commonly used technique to explore the cerebral activities of acupuncture for migraine. fMRI indirectly measures brain activity based on the blood oxygenation level-dependent effect [41], PET-CT relies on the exogenous tracer for measuring the functional and metabolic changes [42], and H-MRS explores the neurochemical changes of the brain [43]. Only one study combing DTI and fMRI to explore the cerebral alterations of acupuncture for migraine [20] ofthe included studies. Multimodal neuroimaging can integrate the strengths of each imaging modality and make the data cross-validation.

Therefore, future studies can combine the multimodal neuroimaging techniques to detect the dynamic brain activity of acupuncture for migraine to make the results complementary and cross-validation. For example, combine molecular imaging such as H-MRS with fMRI to detect cerebral activities from the molecular and functional levels. H-MRS can be a complementary neuroimaging technique to fMRI, which can identify static as well as dynamic levels of specific brain neurotransmitters that likely relate to the fMRI signal involved in pain processing and modulation of acupuncture for migraine [44]. Besides, combing fMRI with electroencephalography can provide complementary information of brain activity (hemodynamic and electrophysiological, respectively) with the spatiotemporal resolution, which can be applied to detect the dynamic cerebral activities of acupuncture for the whole migraine attacks.

### 4.3. Pain Pathways Participating in the Acupuncture for Migraine

The neuroimaging studies about acupuncture for migraine demonstrated that acupuncture modulates a widely distributed network of brain areas including the frontal lobe, temporal lobe, occipital lobe, parietal lobe, diencephalon, brainstem, and cerebellum (Figure 2). According to the high frequency of altered brain regions, the important pain pathways related to the acupuncture for migraine of the included studies were clarified (Figure 2).

#### 4.3.1. The Medial Pain System Participating in the Cerebral Responses to Acupuncture for Migraine

In this review, the included studies have suggested that the regions of the medial pain system, including the anterior cingulate cortex (ACC), insula, and middle frontal gyrus (MFG), are participating in the cerebral responses to acupuncture for migraine (Figure 2(b), orange nodes). The medial pain pathway projects to the limbic system including the ACC and insula via the medial thalamus participating in the process of motivational-affective aspects, cognitive-evaluative aspects, and memory of pain [45, 46]. Chronic pain can lead to persistent emotional disorders such as anxiety, fear, and influence brain processing on many levels [47–49]. Cumulative evidence suggested that ACC contributed to the response to the process of anxiety and fear of pain [50, 51] and insula involved in the processing of the emotional motivation dimension of pain [52, 53]. MFG is often referred to as the dorsolateral prefrontal cortex (dlPFC) [54], dlPFC is a core putative target for modulation of pain-related fear responses [55]. Previous studies suggested that the altered function of the regions of the medial pain system participates in the pathology of migraine [56, 57]. In this review, the key regions of the medial pain system are involved in the modulation of acupuncture for migraine, which indicated that acupuncture may exert its therapeutic efficacy on migraine by modulating the medial pain system.

#### 4.3.2. The Lateral Pain System Participating in the Cerebral Responses to Acupuncture for Migraine

The thalamus cortex is known to be a relay center for the processing of nociceptive inputs, and the thalamus can be divided into medial and ventrobasal parts [58]. Pathways from the medial thalamus project diffusely to wide areas of the cortex and together make up the medial pain system. The ventrobasal thalamus receives input from the ascending tract, sends fibers to the primary and secondary somatosensory areas of the cerebral cortex, and makes up the lateral pain system. The postcentral gyrus (PoCG) was a part of secondary somatosensory areas of the cerebral cortex. The lateral pain system was where refined localization and discrimination of pain occurred. The altered thalamus and PoCG involved in the lateral pain system pointed to abnormal pain processing and modulation in migraine [59–61]. After summarizing the neuroimaging results of acupuncture for migraine, thalamus and PoCG were the high-frequency regions that can be regulated by acupuncture for migraine. Therefore, the lateral pain pathway (Figure 2(b), yellow nodes) processing the localization and discrimination of pain was also the important region of acupuncture for migraine.

Besides, the results indicated that the key regions of default DMN [62] (Figure 2(b), green nodes), including the media prefrontal cortex, cingulate cortex, precuneus, medial temporal lobe, insula, and hippocampus, are involved in the cerebral responses to acupuncture for migraine. DMN is associated with attention, memory, prospection, and self-referential processing [63–65]. Recent studies showed that altered DMN is associated with many chronic pain conditions [66–68], and it is the primary network affected by chronic pain [69]. Previous studies have also found that DMN plays an important role in pain modulation of migraine [70–72]. In this review, most regions of DMN are involved in the modulation of acupuncture for migraine, which indicated that acupuncture may exert its therapeutic effects by modulating the distributed regions for migraine.

#### 4.3.3. The Descending Pain System Participating in the Cerebral Responses to Acupuncture for Migraine

Disruption of the balance of descending pain modulation associated with chronic pain has been confirmed [73, 74]. The well-known descending pain systems majorly contained periaqueductal gray (PAG) and rostral ventromedial medulla (RVM). PAG is the primary region in controlling the modulation of the descending pain modulatory system in the brainstem [75, 76] and affects descending pain modulation primarily through its reciprocal connections with RVM [77]. The descending pain system is significantly implicated in the dysfunction of the descending pain modulatory pathways in migraine [78–80]. The PAG and RVM were activated after the treatment of acupuncture in this review, which suggested that the functions of the descending pain system were not only a response to migraine but also an underlying brain pathway that was perhaps at the core of modulating the pain of migraine.

Further analysis of the relation between the study designs and results indicated that the medial pain pathway was the core altered region during VA vs. SA control modality about the long-term effect [6–8, 12, 13, 16, 26, 27, 33]. The results indicated that the role of the medial pain system, which mainly participated in the pain-related emotional response, was one of the key modulations of acupuncture for migraine. Additionally, the results about adopting one treatment session to explore the immediate-efficacy acupuncture for migraine [9, 10, 12, 14, 18, 19, 21, 26] mainly concentrated on the lateral pain system. The results manifested that the lateral pain system was another key pathway processing the sensation of pain involving the modulation for acupuncture. In other words, the pain experience has a multidimensional nature. The modulation of acupuncture was multimodal rather than unidimensional, which contained the medial pain system for processing the emotion loop, lateral pain system for processing the sensory loop, and descending pain system for inhibiting pain.

## 5. Conclusion

This review mainly overviewed the study design and main findings of acupuncture for migraine by neuroimaging techniques. This review manifested that acupuncture could elicit cerebral responses on patients with migraine and different from SA. Current studies began to use the ML to predict the efficacy of acupuncture for migraine. In the future, researchers can promote the integration of clinical and neuroimaging data with a bigger sample size to predict the efficacy of acupuncture for migraine and provide specific clinical guidance at the individual level.

## Data Availability

The datasets of this study can be requested from the first author at mapeihong@stu.cdutcm.edu.cn.

## Abbreviation

TCM: Traditional Chinese medicine
VA: Verum acupuncture
SA: Sham acupuncture
VAS: Visual analogue scale
MRI: Magnetic resonance imaging
fMRI: Functional magnetic resonance imaging
DTI: Diffusion tensor imaging
PET-CT: Positron emission tomography-computed tomography
H-MRS: Proton magnetic resonance spectroscopy
ALFF/fALFF: Amplitude of low-frequency fluctuations/fractional amplitude of low-frequency fluctuations
ReHo: Regional homogeneity
SPM: Statistical parametric mapping
ML: Machine learning
DMN: Default mode network
MwoA: Migraine without aura
MSQ: Migraine-Specific Quality of Life Questionnaire
QoL: Quality of life
ACC: Anterior cingulate cortex
dlPFC: Dorsolateral prefrontal cortex
PoCG: Postcentral gyrus
MFG: Middle frontal gyrus
PAG: Periaqueductal gray
RVM: Rostral ventromedial medulla.

## Supplementary Materials

Supplementary Materials Supplementary Table 1. Full search strategy for each of the electronic databases queried. Supplementary Table 2. The basic information of the included studies. Supplementary Table 3. The study design of the included studies. Supplementary Table 4. The neuroimage information of the included studies. Supplementary Figure 1. The flow diagram of the literature search and screening process. Supplementary Figure 2. The basic information of the included studies. A. The annual distribution of included studies. B. The institution distribution of included studies.

## References

[1] IHS (2013). The international classification of headache disorders, 3rd edition (beta version). *Cephalalgia*.

[2] Vos T., Allen C., Arora M. (2016). Global, regional, and national incidence, prevalence, and years lived with disability for 310 diseases and injuries, 1990–2015: a systematic analysis for the Global Burden of Disease Study 2015. *Lancet*.

[3] Martelletti P., Birbeck G. L., Katsarava Z., Jensen R. H., Stovner L. J., Steiner T. J. (2013). The global burden of disease survey 2010, lifting the burden and thinking outside-the-box on headache disorders. *The Journal of Headache and Pain*.

[4] Abu Bakar N., Tanprawate S., Lambru G., Torkamani M., Jahanshahi M., Matharu M. (2016). Quality of life in primary headache disorders: a review. *Cephalalgia*.

[5] Buse D. C., Lipton R. B. (2013). Global perspectives on the burden of episodic and chronic migraine. *Cephalalgia*.

[6] Li Z., Zeng F., Yin T. (2017). Acupuncture modulates the abnormal brainstem activity in migraine without aura patients. *NeuroImage: Clinical*.

[7] Li Z., Liu M., Lan L. (2016). Altered periaqueductal gray resting state functional connectivity in migraine and the modulation effect of treatment. *Scientific Reports*.

[8] Li Z., Lan L., Zeng F. (2017). The altered right frontoparietal network functional connectivity in migraine and the modulation effect of treatment. *Cephalalgia*.

[9] Li X., Liu X., Song W., Tang Y., Zeng F., Liang F. (2008). Effect of acupuncture at acupoints of the Shaoyang Meridian on cerebral glucose metabolism in the patient of chronic migraine (Chinese Version). *Chinese Acupuncture & Moxibustion*.

[10] Li X., Liu X., Song W. (2008). Effect of acupuncture on cerebral glucose metabolism in chronic migraineurs: a PET-CT study (Chinese version). *Journal of Chengdu University of TCM*.

[11] Zhao L., Liu J., Zhang F. (2014). Effects of long-term acupuncture treatment on resting-state brain activity in migraine patients: a randomized controlled trial on active acupoints and inactive acupoints. *PLoS One*.

[12] Yang M., Yang J., Zeng F. (2014). Electroacupuncture stimulation at sub-specific acupoint and non-acupoint induced distinct brain glucose metabolism change in migraineurs: a PET-CT study. *Journal of Translational Medicine*.

[13] Zhang Y., Xu T., Wang X., Wang Z., Du J., Zhao L. (2020). Exploration on the effects of acupuncture on the precuneus functional connectivity of menstrual migraine patients by fMRI (Chinese Version). *China Journal of Traditional Chinese Medicine and Pharmacy*.

[14] Yang J., Zeng F., Feng Y. (2012). A PET-CT study on the specificity of acupoints through acupuncture treatment in migraine patients. *BMC Complementary and Alternative Medicine*.

[15] Yin T., Sun G., Tian Z. (2020). The spontaneous activity pattern of the middle occipital gyrus predicts the clinical efficacy of acupuncture treatment for migraine without aura. *Frontiers in Neurology*.

[16] Tu Y., Zeng F., Lan L. (2020). An fMRI-based neural marker for migraine without aura. *Neurology*.

[17] Wu K., Xu L., Li K. (2020). Effect of acupuncture on structual brain network in patients with different course of disease migraine without aura (Chinese Version). *Journal of Traditional Chinese Medicine*.

[18] Han X., Zou Y., Li K. (2017). Effect of acupunture at GB41 on migraine patients on the cortical regional homogeneity (ReHo) in the patient of chronic migraine (Chinese Version). *Modern Chinese Clinical Medicine*.

[19] Liu H., Li K., Ning Y. (2016). Effects of acupuncture at Zulinqi (GB41) on pain related brain networks of migraine patients: an fMRI study (Chinese Version). *China Journal of Traditional Chinese Medicine and Pharmacy*.

[20] Li K., Zhang Y., Ning Y. (2015). The effects of acupuncture treatment on the right frontoparietal network in migraine without aura patients. *The Journal of Headache and Pain*.

[21] Ning Y., Zheng R., Lv Y., Fu C., Liu H., Ren Y. (2020). Study on the influence of acupuncture zulinqi (GB41) on the amplitude of low frequency oscillation of migraine (Chinese version). *World Chinese Medicine*.

[22] Liu J., Mu J., Chen T., Zhang M., Tian J. (2019). White matter tract microstructure of the mPFC‐amygdala predicts interindividual differences in placebo response related to treatment in migraine patients. *Human Brain Mapping*.

[23] Liu J., Ma S., Mu J. (2017). Integration of white matter network is associated with interindividual differences in psychologically mediated placebo response in migraine patients. *Human Brain Mapping*.

[24] Liu J., Mu J., Liu Q., Dun W., Zhang M., Tian J. (2017). Brain structural properties predict psychologically mediated hypoalgesia in an 8-week sham acupuncture treatment for migraine. *Human Brain Mapping*.

[25] Chen X., Lin X., Xu X., Wu J. (2019). Effect of acupuncture at acupoints of the Shaoyang Meridian on diffusion tensor imaging in the patient of chronic migraine (Chinese Version). *Chinese Journal of Integrative Medicine on Cardio-Cerebrovascular Disease*.

[26] Luo W., Zhang Y., Zhang Y., Zhou S., Yan Z., Liu B. (2019). Effect of auricular acupoint continuous stimulation on brain fraction amplitude of low-frequency fluctuation in patients with migraine without aura (Chinese Version). *Chinese Imaging Journal of Integrated Traditional and Western Medicine*.

[27] Tan X., Wang W., Wang J., Xie W., Zhang Y., Gao Y. (2019). Analysis on regional homogeneity of resting brain during balance acupuncture-induced analgesic effect in migraine patients without aura (Chinese Version). *Acupuncture Research*.

[28] Zhang Y., Li K.-S., Liu H.-W. (2016). Acupuncture treatment modulates the resting-state functional connectivity of brain regions in migraine patients without aura. *Chinese Journal of Integrative Medicine*.

[29] Zou Y., Tang W., Li X., Xu M., Li J. (2019). Acupuncture reversible effects on altered default mode network of chronic migraine accompanied with clinical symptom relief. *Neural Plasticity*.

[30] Gu T., Lin L., Jiao S., Ding R. (2013). Effect of acupuncture prophylaxis on cerebral metabolism in migraine: a magnetic resonance spectroscopy study (Chinese Version). *Journal of Medical Imaging*.

[31] Lin L., Gu T., Ding R. (2013). Effect of acupuncture prophylaxis on left thalamus metabolism in migraine: a magnetic resonance spectroscopy study (Chinese Version). *Chinese Journal of Clinical Hepatology*.

[32] Lin L., Ding R., Gu T. (2015). Effect of elecacupuncture on right thalamus and anterior cingulate gyrus Metabolism in Migraine: a magnetic resonance spectroscopy study (Chinese Version). *Journal of Traditional Chinese Medicine*.

[33] Liang R., Zhang S., Xie Y. (2016). Study about influence of brain metabolism in patients with chronic migraine after acupuncture at shaoyang specific acupoints (Chinese version). *Chinese Archives of Traditional Chinese Medicine*.

[34] Marciszewski K. K., Meylakh N., Di Pietro F. (2018). Changes in brainstem pain modulation circuitry function over the migraine cycle. *Journal of Neuroscience*.

[35] Stankewitz A., Aderjan D., Eippert F., May A. (2011). Trigeminal nociceptive transmission in migraineurs predicts migraine attacks. *Journal of Neuroscience*.

[36] Tedeschi G., Russo A., Conte F., Salemi F., Tessitore A. (2013). The role of BOLD-fMRI in elucidating migraine pathophysiology. *Neurological Sciences: Official Journal of the Italian Neurological Society and of the Italian Society of Clinical Neurophysiology*.

[37] Pope N., Tallon M., McConigley R., Wilson S. (2015). The experiences of acute non-surgical pain of children who present to a healthcare facility for treatment: a systematic review protocol. *JBI database of systematic reviews and implementation reports*.

[38] Peres M. F. P., Mercante J. P. P., Tobo P. R., Kamei H., Bigal M. E. (2017). Anxiety and depression symptoms and migraine: a symptom-based approach research. *The Journal of Headache and Pain*.

[39] Holroyd K., Drew J., Cottrell C., Romanek K., Heh V. (2007). Impaired functioning and quality of life in severe migraine: the role of catastrophizing and associated symptoms. *Cephalalgia*.

[40] Dworkin R. H., Turk D. C., Wyrwich K. W. (2008). Interpreting the clinical importance of treatment outcomes in chronic pain clinical trials: IMMPACT recommendations. *The Journal of Pain*.

[41] Pidnebesna A., Tomeček D., Hlinka J. (2018). BRAD: software for BRain activity detection from hemodynamic response. *Computer Methods and Programs in Biomedicine*.

[42] Ogawa S., Lee T. M., Kay A. R., Tank D. W. (1990). Brain magnetic resonance imaging with contrast dependent on blood oxygenation. *Proceedings of the National Academy of Sciences*.

[43] Borsook D., Hargreaves R. (2010). Brain imaging in migraine research. *Headache: The Journal of Head and Face Pain*.

[44] Napadow V., Harris R. E. (2014). What has functional connectivity and chemical neuroimaging in fibromyalgia taught us about the mechanisms and management of ‘centralized’ pain?. *Arthritis Research and Therapy*.

[45] Vogt B. A., Sikes R. W. (2000). The medial pain system, cingulate cortex, and parallel processing of nociceptive information. *The Biological Basis for Mind Body Interactions*.

[46] Sewards T. V., Sewards M. A. (2002). The medial pain system: neural representations of the motivational aspect of pain. *Brain Research Bulletin*.

[47] Wise R. G., Lujan B. J., Schweinhardt P., Peskett G. D., Rogers R., Tracey I. (2007). The anxiolytic effects of midazolam during anticipation to pain revealed using fMRI. *Magnetic Resonance Imaging*.

[48] Zhuo M. (2008). Cortical excitation and chronic pain. *Trends in Neurosciences*.

[49] Kain Z. N., Mayes L. C., Caldwell-Andrews A. A., Karas D. E., McClain B. C. (2006). Preoperative anxiety, postoperative pain, and behavioral recovery in young children undergoing surgery. *Pediatrics*.

[50] Yoshino A., Okamoto Y., Onoda K. (2010). Sadness enhances the experience of pain via neural activation in the anterior cingulate cortex and amygdala: an fMRI study. *NeuroImage*.

[51] Bliss T. V. P., Collingridge G. L., Kaang B.-K., Zhuo M. (2016). Synaptic plasticity in the anterior cingulate cortex in acute and chronic pain. *Nature Reviews Neuroscience*.

[52] Treede R. D., Kenshalo D. R., Gracely R. H., Jones A. K. (1999). The cortical representation of pain. *Pain*.

[53] Wang N., Zhang Y. H., Wang J. Y., Luo F. (2021). Current understanding of the involvement of the insular cortex in neuropathic pain: a narrative review. *International Journal of Molecular Sciences*.

[54] Fullana M. A., Harrison B. J., Soriano-Mas C. (2016). Neural signatures of human fear conditioning: an updated and extended meta-analysis of fMRI studies. *Molecular Psychiatry*.

[55] Seminowicz D. A., Moayedi M. (2017). The dorsolateral prefrontal cortex in acute and chronic pain. *The Journal of Pain*.

[56] Xue T., Yuan K., Cheng P. (2013). Alterations of regional spontaneous neuronal activity and corresponding brain circuit changes during resting state in migraine without aura. *NMR in Biomedicine*.

[57] Zhang J., Su J., Wang M. (2017). The sensorimotor network dysfunction in migraineurs without aura: a resting-state fMRI study. *Journal of Neurology*.

[58] Caston R. M., Smith E. H., Davis T. S., Rolston J. D. (2020). The cerebral localization of pain: anatomical and functional considerations for targeted electrical therapies. *Journal of Clinical Medicine*.

[59] Younis S., Hougaard A., Noseda R., Ashina M. (2019). Current understanding of thalamic structure and function in migraine. *Cephalalgia*.

[60] Bahra A., Matharu M., Buchel C., Frackowiak R., Goadsby P. (2001). Brainstem activation specific to migraine headache. *The Lancet*.

[61] Afridi S. K., Matharu M. S., Lee L. (2005). A PET study exploring the laterality of brainstem activation in migraine using glyceryl trinitrate. *Brain: A Journal of Neurology*.

[62] Raichle M. E. (2015). The brain’s default mode network. *Annual Review of Neuroscience*.

[63] Eccleston C., Crombez G. (1999). Pain demands attention: a cognitive-affective model of the interruptive function of pain. *Psychological Bulletin*.

[64] Raichle M. E., Snyder A. Z. (2007). A default mode of brain function: a brief history of an evolving idea. *NeuroImage*.

[65] Buckner R. L., Andrews-Hanna J. R., Schacter D. L. (2008). The brain’s default network. *Annals of the New York Academy of Sciences*.

[66] Tagliazucchi E., Balenzuela P., Fraiman D., Chialvo D. R. (2010). Brain resting state is disrupted in chronic back pain patients. *Neuroscience Letters*.

[67] Napadow V., Kim J., Clauw D. J., Harris R. E. (2012). Brief report: decreased intrinsic brain connectivity is associated with reduced clinical pain in fibromyalgia. *Arthritis & Rheumatism*.

[68] Letzen J. E., Craggs J. G., Perlstein W. M., Price D. D., Robinson M. E. (2013). Functional connectivity of the default mode network and its association with pain networks in irritable bowel patients assessed via lidocaine treatment. *The Journal of Pain*.

[69] Farmer M. A., Baliki M. N., Apkarian A. V. (2012). A dynamic network perspective of chronic pain. *Neuroscience Letters*.

[70] Xue T., Yuan K., Zhao L. (2012). Intrinsic brain network abnormalities in migraines without aura revealed in resting-state fMRI. *PLoS One*.

[71] Tessitore A., Russo A., Giordano A. (2013). Disrupted default mode network connectivity in migraine without aura. *The Journal of Headache and Pain*.

[72] Hubbard C. S., Khan S. A., Keaser M. L., Mathur V. A., Goyal M., Seminowicz D. A. (2014). Altered brain structure and function correlate with disease severity and pain catastrophizing in migraine patients. *eNeuro*.

[73] Chen Q., Heinricher M. M. (2019). Descending control mechanisms and chronic pain. *Current Rheumatology Reports*.

[74] Ossipov M. H., Morimura K., Porreca F. (2014). Descending pain modulation and chronification of pain. *Current Opinion in Supportive & Palliative Care*.

[75] Yaksh T. L., Tyce G. M. (1979). Microinjection of morphine into the periaqueductal gray evokes the release of serotonin from spinal cord. *Brain Research*.

[76] Apkarian A. V., Baliki M. N., Geha P. Y. (2009). Towards a theory of chronic pain. *Progress in Neurobiology*.

[77] Willis W. D. (1985). Central nervous system mechanisms for pain modulation. *Applied Neurophysiology*.

[78] Schwedt T. J., Larson-Prior L., Coalson R. S. (2014). Allodynia and descending pain modulation in migraine: a resting state functional connectivity analysis. *Pain Medicine*.

[79] Mainero C., Boshyan J., Hadjikhani N. (2011). Altered functional magnetic resonance imaging resting-state connectivity in periaqueductal gray networks in migraine. *Annals of Neurology*.

[80] Holland P. (2009). Modulation of trigeminovascular processing: novel insights into primary headache disorders. *Cephalalgia*.

