# Supplementary material for: A Narrative Review of Neuroimaging Studies in Acupuncture for Migraine
Source: Pain Res Manag. 2021 Nov 10;2021:9460695. doi: 10.1155/2021/9460695 (PMC8598357; doi:10.1155/2021/9460695)
Supplement: Supplementary Materials — Supplementary Table 1. Full search strategy for each of the electronic databases queried. Supplementary Table 2. The basic information of the included studies. Supplementary Table 3. The study design of the included studies. Supplementary Table 4. The neuroimage information of the included studies. Supplementary Figure 1. The flow diagram of the literature search and screening process. Supplementary Figure 2. The basic information of the included studies. A. The annual distribution of included studies. B. The institution distribution of included studies. [file 9460695.f1.zip › Revised_Supplementary Table_2_basic_information_reference.docx]

**Supplementary Table. 2. The Basic Information of the Included Studies.**

| Study  No. | Title | Publish Time | Institution | Trial place | Journal | Language of Publication |
| --- | --- | --- | --- | --- | --- | --- |
| S01(1) | The Spontaneous Activity Pattern of the Middle Occipital Gyrus Predicts the Clinical Efficacy of Acupuncture Treatment for Migraine Without Aura | 2020 | Chengdu University of Traditional Chinese Medicine | China | Frontiers in Neurology | ENG |
| S02(2) | An fMRI-based neural marker for migraine without aura | 2020 | Chengdu University of Traditional Chinese Medicine | China | Neurology | ENG |
| S03(3) | Exploration on the effects of acupuncture on the precuneus functional connectivity of menstrual migraine patients by fMRI (Chinese Version) | 2020 | Chengdu University of Traditional Chinese Medicine | China | China Journal of Traditional Chinese Medicine and Pharmacy | CHN |
| S04(4) | Acupuncture modulates the abnormal brainstem activity in migraine without aura patients | 2017 | Chengdu University of Traditional Chinese Medicine | China | Neurolmage Clinical | ENG |
| S05(5) | The altered right frontoparietal network functional connectivity in migraine and the modulation effect of treatment | 2017 | Chengdu University of Traditional Chinese Medicine | China | Cephalalgia | ENG |
| S06(6) | Altered periaqueductal gray resting state functional connectivity in migraine and the modulation effect of treatment | 2016 | Chengdu University of Traditional Chinese Medicine | China | Scientific Reports | ENG |
| S07(7) | Effects of Long-Term Acupuncture Treatment on Resting-State Brain Activity in Migraine Patients: A Randomized Controlled Trial on Active Acupoints and Inactive Acupoints | 2014 | Chengdu University of Traditional Chinese Medicine | China | PLOS One | ENG |
| S08(8) | Electroacupuncture stimulation at sub-specific acupoint and non-acupoint induced distinct brain glucose metabolism change in migraineurs: a PET-CT study | 2014 | Chengdu University of Traditional Chinese Medicine | China | Journal of Translational Medicine | ENG |
| S09(9) | A PET-CT study on the specificity of acupoints through acupuncture treatment in migraine patients | 2012 | Chengdu University of Traditional Chinese Medicine | China | BMC Complementary and Alternative Medicine | ENG |
| S10(10) | Effect of acupuncture at acupoints of the Shaoyang Meridian on cerebral glucose metabolism in the patient of chronic migraine (Chinese Version) | 2008 | Chengdu University of Traditional Chinese Medicine | China | Chinese Acupuncture & Moxibustion | CHN |
| S11(11) | Effect of Acupuncture on Cerebral Glucose Metabolism in Chronic Migraineurs: A PET-CT Study (Chinese Version) | 2008 | Chengdu University of Traditional Chinese Medicine | China | Journal of Chengdu University of TCM | CHN |
| S12(12) | Study on the Influence of Acupuncture Zulinqi(GB41)on the Amplitude of Low Frequency Oscillation of Migraine (Chinese Version) | 2020 | Dongzhimen Hospital of Beijing University of Chinese Medicine | China | World Chinese Medicine | CHN |
| S13(13) | Effect of acupuncture on structural brain network in patients with different course of disease migraine without aura (Chinese Version) | 2020 | Dongzhimen Hospital of Beijing University of Chinese Medicine | China | Journal of Traditional Chinese Medicine | CHN |
| S14(14) | Effect of acupuncture at GB41 on migraine patients on the cortical regional homogeneity (ReHo) in the patient of chronic migraine (Chinese Version) | 2017 | Dongzhimen Hospital of Beijing University of Chinese Medicine | China | Modern Chinese Clinical Medicine | CHN |
| S15(15) | Effects of acupuncture at Zulinqi(GB41) on pain related brain networks of migraine patients: An fMRI study (Chinese Version) | 2016 | Dongzhimen Hospital of Beijing University of Chinese Medicine | China | China Journal of Traditional Chinese Medicine and Pharmacy | CHN |
| S16(16) | The effects of acupuncture treatment on the right frontoparietal network in migraine without aura patients | 2015 | Dongzhimen Hospital of Beijing University of Chinese Medicine | China | The journal of headache and pain | ENG |
| S17(17) | White matter tract microstructure of the mPFC-amygdala predicts interindividual differences in placebo response related to treatment in migraine patients | 2018 | Xidian university | China | Human Brain Mapping | ENG |
| S18(18) | Brain structural properties predict psychologically mediated hypoalgesia in an 8-week sham acupuncture treatment for migraine | 2017 | Xidian University | China | Human Brain Mapping | ENG |
| S19(19) | Integration of white matter network is associated with interindividual differences in psychologically mediated placebo response in migraine patients | 2017 | Xidian University | China | Human Brain Mapping | ENG |
| S20(20) | Effect of electroacupuncture on right thalamus and anterior cingulate gyrus Metabolism in Migraine: a magnetic resonance spectroscopy study (Chinese Version) | 2015 | Beijing Hospital | China | Journal of Traditional Chinese Medicine | CHN |
| S21(21) | Effect of acupuncture prophylaxis on left thalamus metabolism in migraine: a magnetic resonance spectroscopy study (Chinese Version) | 2013 | Beijing Hospital | China | Chinese Journal of Clinical Healthcare | CHN |
| S22(22) | Effect of acupuncture prophylaxis on cerebral metabolism in migraine: a magnetic resonance spectroscopy study (Chinese Version) | 2013 | Beijing Hospital | China | Journal of Medical Imaging | CHN |
| S23(23) | Effect of auricular acupoint continuous stimulation on brain fraction amplitude of low-frequency fluctuation in patients with migraine without aura (Chinese Version) | 2019 | Second Affiliated Hospital of Guangzhou University of Chinese Medicine | China | Chinese Imaging Journal of Integrated Traditional and Western Medicine | CHN |
| S24(24) | Analysis on regional homogeneity of resting brain during balance acupuncture-induced analgesic effect in migraine patients without aura (Chinese Version) | 2019 | Second Affiliated Hospital of Guangzhou University of Chinese Medicine | China | Acupuncture Research | CHN |
| S25(25) | Acupuncture Reversible Effects on Altered Default Mode Network of Chronic Migraine Accompanied with Clinical Symptom Relief | 2019 | Fudan University | China | Neural Plasticity | ENG |
| S26(26) | Acupuncture Treatment Modulates the Resting-State Functional Connectivity of Brain Regions in migraine patients without aura | 2016 | First Affiliated Hospital of Beijing University of Chinese Medicine | China | Chinese journal of integrative medicine | ENG |
| S27(27) | Study about Influence of Brain Metabolism in Patients with Chronic Migraine after Acupuncture at Shaoyang Specific Acupoints (Chinese Version) | 2016 | Fourth Hospital Affiliated to Jinan University | China | Chinese Archives of Traditional Chinese Medicine | CHN |
| S28(28) | Effect of acupuncture at acupoints of the Shaoyang Meridian on diffusion tensor imaging in the patient of chronic migraine (Chinese Version) | 2019 | Peking University Shenzhen Hospital | China | Chinese Journal of Integrative Medicine on Cardio-Cerebrovascular Disease | CHN |

**Reference**

1. Yin T, Sun G, Tian Z, Liu M, Gao Y, Dong M, et al. The Spontaneous Activity Pattern of the Middle Occipital Gyrus Predicts the Clinical Efficacy of Acupuncture Treatment for Migraine Without Aura. Front Neurol. 2020;11:588207.

2. Tu Y, Zeng F, Lan L, Li Z, Maleki N, Liu B, et al. An fMRI-based neural marker for migraine without aura. Neurology. 2020;94(7):e741-e51.

3. Zhang Y, Xu T, Wang X, Wang Z, Du J, Zhao L. Exploration on the effects of acupuncture on the precuneus functional connectivity of menstrual migraine patients by fMRI (Chinese Version). China Journal of Traditional Chinese Medicine and Pharmacy. 2020;35(02):1002-6.

4. Li Z, Zeng F, Yin T, Lan L, Makris N, Jorgenson K, et al. Acupuncture modulates the abnormal brainstem activity in migraine without aura patients. Neuroimage Clin. 2017;15:367-75.

5. Li Z, Lan L, Zeng F, Makris N, Hwang J, Guo T, et al. The altered right frontoparietal network functional connectivity in migraine and the modulation effect of treatment. Cephalalgia. 2017;37(2):161-76.

6. Li Z, Liu M, Lan L, Zeng F, Makris N, Liang Y, et al. Altered periaqueductal gray resting state functional connectivity in migraine and the modulation effect of treatment. Sci Rep. 2016;6:20298.

7. Zhao L, Liu J, Zhang F, Dong X, Peng Y, Qin W, et al. Effects of long-term acupuncture treatment on resting-state brain activity in migraine patients: a randomized controlled trial on active acupoints and inactive acupoints. PLoS One. 2014;9(6):e99538.

8. Yang M, Yang J, Zeng F, Liu P, Lai Z, Deng S, et al. Electroacupuncture stimulation at sub-specific acupoint and non-acupoint induced distinct brain glucose metabolism change in migraineurs: a PET-CT study. J Transl Med. 2014;12:351.

9. Yang J, Zeng F, Feng Y, Fang L, Qin W, Liu X, et al. A PET-CT study on the specificity of acupoints through acupuncture treatment in migraine patients. BMC Complement Altern Med. 2012;12:123.

10. Li X, Liu X, Song W, Tang Y, Zeng F, Liang F. Effect of acupuncture at acupoints of the Shaoyang Meridian on cerebral glucose metabolism in the patient of chronic migraine (Chinese Version). Chinese Acupuncture & Moxibustion. 2008(11):854-9.

11. Li X, Liu X, Song W, Tang Y, Gao H, Zeng F, et al. Effect of Acupuncture on Cerebral Glucose Metabolism in Chronic Migraineurs: A PET-CT Study (Chinese Version). Journal of Chengdu University of TCM. 2008(03):1-5.

12. Ning Y, Zheng R, Lv Y, Fu C, Liu H, Ren Y. Study on the Influence of Acupuncture Zulinqi(GB41)on the Amplitude of Low Frequency Oscillation of Migraine (Chinese Version). World Chinese Medicine. 2020;15(20):3131-7.

13. Wu K, Xu L, Li K, Ren Y, Zou Y, Jiang L, et al. Effect of acupuncture on structual brain network in patients with different course of disease migraine without aura (Chinese Version). Journal of Traditional Chinese Medicine. 2020;61(24):2184-9.

14. Han X, Zou Y, Li K, Liu H, Ning Y, Tan Z, et al. Effect of acupunture at GB41 on migraine patients on the cortical regional homogeneity (ReHo) in the patient of chronic migraine (Chinese Version). Modern Chinese Clinical Medicine. 2017;24(06):31-5+65.

15. Liu H, Li K, Ning Y, Han X, Tan Z, Ren Y, et al. Effects of acupuncture at Zulinqi(GB41) on pain related brain networks of migraine patients: An fMRI study (Chinese Version). China Journal of Traditional Chinese Medicine and Pharmacy. 2016;31(05):2013-6.

16. Li K, Zhang Y, Ning Y, Zhang H, Liu H, Fu C, et al. The effects of acupuncture treatment on the right frontoparietal network in migraine without aura patients. J Headache Pain. 2015;16:518.

17. Liu J, Mu J, Chen T, Zhang M, Tian J. White matter tract microstructure of the mPFC-amygdala predicts interindividual differences in placebo response related to treatment in migraine patients. Hum Brain Mapp. 2019;40(1):284-92.

18. Liu J, Mu J, Liu Q, Dun W, Zhang M, Tian J. Brain structural properties predict psychologically mediated hypoalgesia in an 8-week sham acupuncture treatment for migraine. Hum Brain Mapp. 2017;38(9):4386-97.

19. Liu J, Ma S, Mu J, Chen T, Xu Q, Dun W, et al. Integration of white matter network is associated with interindividual differences in psychologically mediated placebo response in migraine patients. Hum Brain Mapp. 2017;38(10):5250-9.

20. Lin L, Ding R, Gu T. Effect of elecacupuncture on right thalamus and anterior cingulate gyrus Metabolism in Migraine: a magnetic resonance spectroscopy study (Chinese Version). Journal of Traditional Chinese Medicine. 2015;56(14):1220-3.

21. Lin L, Gu T, Ding R. Effect of acupuncture prophylaxis on left thalamus metabolism in migraine: a magnetic resonance spectroscopy study (Chinese Version). Chinese Journal of Clinical Healthcare. 2013;16(02):190-2.

22. Gu T, Lin L, Jiao S, Ding R. Effect of acupuncture prophylaxis on cerebral metabolism in migraine: a magnetic resonance spectroscopy study (Chinese Version). Journal of Medical Imaging. 2013;23(03):345-9.

23. Luo W, Zhang Y, Zhang Y, Zhou S, Yan Z, Liu B. Effect of auricular acupoint continuous stimulation on brain fraction amplitude of low-frequency fluctuation in patients with migraine without aura (Chinese Version). Chinese Imaging Journal of Integrated Traditional and Western Medicine. 2019;17(05):441-4.

24. Tan X, Wang W, Wang J, Xie W, Zhang Y, Gao Y. Analysis on regional homogeneity of resting brain during balance acupuncture-induced analgesiceffect in migraine patients without aura (Chinese Version). Acupuncture Research. 2019;44(06):446-50.

25. Zou Y, Tang W, Li X, Xu M, Li J. Acupuncture Reversible Effects on Altered Default Mode Network of Chronic Migraine Accompanied with Clinical Symptom Relief. Neural Plast. 2019;2019:5047463.

26. Zhang Y, Li KS, Liu HW, Fu CH, Chen S, Tan ZJ, et al. Acupuncture treatment modulates the resting-state functional connectivity of brain regions in migraine patients without aura. Chin J Integr Med. 2016;22(4):293-301.

27. Liang R, Zhang S, Xie Y. Study about Influence of Brain Metabolism in Patients with Chronic Migraine after Acupuncture at Shaoyang Specific Acupoints (Chinese Version). Chinese Archives of Traditional Chinese Medicine. 2016;34(04):918-20.

28. Chen X, Lin X, Xu X, Wu J. Effect of acupuncture at acupoints of the Shaoyang Meridian on diffusion tensor imaging in the patient of chronic migraine (Chinese Version). Chinese Journal of Integrative Medicine on Cardio-Cerebrovascular Disease. 2019;17(07):1092-3.
