# Supplementary material for: A Narrative Review of Neuroimaging Studies in Acupuncture for Migraine
Source: Pain Res Manag. 2021 Nov 10;2021:9460695. doi: 10.1155/2021/9460695 (PMC8598357; doi:10.1155/2021/9460695)
Supplement: Supplementary Materials — Supplementary Table 1. Full search strategy for each of the electronic databases queried. Supplementary Table 2. The basic information of the included studies. Supplementary Table 3. The study design of the included studies. Supplementary Table 4. The neuroimage information of the included studies. Supplementary Figure 1. The flow diagram of the literature search and screening process. Supplementary Figure 2. The basic information of the included studies. A. The annual distribution of included studies. B. The institution distribution of included studies. [file 9460695.f1.zip › Revised_Supplementary Table_3_study_design_reference.docx]

**Supplementary Table. 3. The Study Design of the Included Studies.**

| Study  No. | Patients | Intervention | | | | Controls | Clinical variables |
| --- | --- | --- | --- | --- | --- | --- | --- |
|  |  | **Manipulation** | **Course of Treatments** | | |  |  |
|  |  |  | **Time** | **Sessions** | **Frequency** |  |  |
| S01([1](#_ENREF_1)) | Migraine Without Aura | MA | 30min | 20 | 5t/w*4w | Pre- vs Pos-Treatment | VAS; Migraine Days |
| S02([2](#_ENREF_2)) | Migraine Without Aura | MA | 30min | 20 | 5t/w*4w | VA vs SA | Frequency of Migraine Attacks |
| S03([3](#_ENREF_3)) | Menstrual Migraine | MA | 30min | 27 or (27±6) | 7t/m*3m | VA vs SA | VAS; Frequency of Migraine Attacks; Migraine Intensity |
| S04([4](#_ENREF_4)) | Migraine Without Aura | EA | 30min | 20 | 5t/w*4w | VA vs SA | VAS; Frequency of Migraine Attacks |
| S05([5](#_ENREF_5)) | Migraine Without Aura | MA | 30min | 20 | 5t/w*4w | VA vs SA | VAS; Frequency of Migraine Attacks |
| S06([6](#_ENREF_6)) | Migraine Without Aura | MA | 30min | 20 | 5t/w*4w | VA vs SA | VAS; Frequency of Migraine Attacks |
| S07([7](#_ENREF_7)) | Migraine Without Aura | EA | 30min | 32 | 4t/w*8w | Different Acupoints | VAS; Migraine Days; Frequency of Migraine Attacks |
| S08([8](#_ENREF_8)) | Migraine | EA | 30min | 1 | 1 | VA vs SA | VAS |
| S09([9](#_ENREF_9)) | Migraine Without Aura | EA | 30min | 1 | 1 | Different Acupoints | VAS |
| S10([10](#_ENREF_10)) | Chronic Migraine | MA | 30min | 1 | 1 | Pre- vs Pos-Treatment | No Depicted |
| S11([11](#_ENREF_11)) | Chronic Migraine | MA | 30min | 1 | 1 | Pre- vs Pos-Treatment | No Depicted |
| S12([12](#_ENREF_12)) | Migraine Without Aura | MA | 9min | 1 | 1 | Pre- vs Pos-Treatment | No Depicted |
| S13([13](#_ENREF_13)) | Migraine Without Aura | MA | 30min | 12 | 3t/w*4w | Different Courses of Migraine | VAS |
| S14([14](#_ENREF_14)) | Migraine Without Aura | MA | 9min | 1 | 1 | Pre- vs Pos-Treatment | No Depicted |
| S15([15](#_ENREF_15)) | Migraine Without Aura | MA | 9min | 1 | 1 | Pre- vs Pos-Treatment | No Depicted |
| S16([16](#_ENREF_16)) | Migraine Without Aura | MA | 30min | 20 | 5t/w*4w | Pre- vs Pos-Treatment | VAS; Frequency of migraine attacks; Migraine Attack Duration; |
| S17([17](#_ENREF_17)) | Migraine Without Aura | MA | 30min | 24 | 3t/w*8w | Different Condition of Migraine (Recovering vs Persisting Migraine) | VAS; Migraine Days; Migraine Attack Duration; MSQ |
| S18([18](#_ENREF_18)) | Migraine Without Aura | MA | 30min | 24 | 3t/w*8w | Different Condition of Migraine (Recovering vs Persisting Migraine) | VAS; Migraine Days; Migraine Attack Duration |
| S19([19](#_ENREF_19)) | Migraine Without Aura | MA | 30min | 24 | 3t/w*8w | Different Condition of Migraine (Recovering vs Persisting Migraine) | VAS; Migraine Days; Migraine Attack Duration |
| S20([20](#_ENREF_20)) | Migraine | EA | 30min | 5 | 1t/d*5d | Different Condition of Migraine (Migraine vs Headache) | No Depicted |
| S21([21](#_ENREF_21)) | Migraine | EA | 30min | 5 | 1t/d*5d | Different Condition of Migraine (Migraine vs Headache) | Migraine Attack Duration; Migraine Intensity |
| S22([22](#_ENREF_22)) | Migraine | EA | 30min | 5 | 1t/d*5d | Different Condition of Migraine (Migraine vs Headache) | Migraine Attack Duration; Migraine Intensity |
| S23([23](#_ENREF_23)) | Migraine Without Aura | Auricular acupoints continuous stimulation | 8min | 1 | 1 | VA vs SA | No Depicted |
| S24([24](#_ENREF_24)) | Migraine Without Aura | MA | 1min | 28 | 1t/d*28d | VA vs SA | VAS |
| S25([25](#_ENREF_25)) | Migraine Without Aura | MA | 30min | 36 | 3t/w*3m | Pre- vs Pos-Treatment | VAS; Migraine attacks, Migraine days |
| S26([26](#_ENREF_26)) | Migraine Without Aura | MA | 30min | 20 | 5t/w*4w | Pre- vs Pos-Treatment | VAS; Frequency of Migraine Attacks; Migraine Attack Duration |
| S27([27](#_ENREF_27)) | Chronic Migraine | MA | 30min | 20 | 5t/w*4w | VA vs SA | VAS |
| S28([28](#_ENREF_28)) | Migraine Without Aura | EA | 20min | 20 | 5t/w*4w | Different Acupuncture Intensities | MSQ |

**Abbr.**

VA. Verum Acupuncture; SA. Sham Acupuncture;

MA. Manual Acupuncture; EA. Electroacupuncture;

d. day; m. month; w. week; t. time

VAS. Visual Analogue Scale; MSQ. Migraine Specific Quality of Life Questionnaire;

**Reference.**

1. Yin T, Sun G, Tian Z, Liu M, Gao Y, Dong M, et al. The Spontaneous Activity Pattern of the Middle Occipital Gyrus Predicts the Clinical Efficacy of Acupuncture Treatment for Migraine Without Aura. Front Neurol. 2020;11:588207.

2. Tu Y, Zeng F, Lan L, Li Z, Maleki N, Liu B, et al. An fMRI-based neural marker for migraine without aura. Neurology. 2020;94(7):e741-e51.

3. Zhang Y, Xu T, Wang X, Wang Z, Du J, Zhao L. Exploration on the effects of acupuncture on the precuneus functional connectivity of menstrual migraine patients by fMRI (Chinese Version). China Journal of Traditional Chinese Medicine and Pharmacy. 2020;35(02):1002-6.

4. Li Z, Zeng F, Yin T, Lan L, Makris N, Jorgenson K, et al. Acupuncture modulates the abnormal brainstem activity in migraine without aura patients. Neuroimage Clin. 2017;15:367-75.

5. Li Z, Lan L, Zeng F, Makris N, Hwang J, Guo T, et al. The altered right frontoparietal network functional connectivity in migraine and the modulation effect of treatment. Cephalalgia. 2017;37(2):161-76.

6. Li Z, Liu M, Lan L, Zeng F, Makris N, Liang Y, et al. Altered periaqueductal gray resting state functional connectivity in migraine and the modulation effect of treatment. Sci Rep. 2016;6:20298.

7. Zhao L, Liu J, Zhang F, Dong X, Peng Y, Qin W, et al. Effects of long-term acupuncture treatment on resting-state brain activity in migraine patients: a randomized controlled trial on active acupoints and inactive acupoints. PLoS One. 2014;9(6):e99538.

8. Yang M, Yang J, Zeng F, Liu P, Lai Z, Deng S, et al. Electroacupuncture stimulation at sub-specific acupoint and non-acupoint induced distinct brain glucose metabolism change in migraineurs: a PET-CT study. J Transl Med. 2014;12:351.

9. Yang J, Zeng F, Feng Y, Fang L, Qin W, Liu X, et al. A PET-CT study on the specificity of acupoints through acupuncture treatment in migraine patients. BMC Complement Altern Med. 2012;12:123.

10. Li X, Liu X, Song W, Tang Y, Zeng F, Liang F. Effect of acupuncture at acupoints of the Shaoyang Meridian on cerebral glucose metabolism in the patient of chronic migraine (Chinese Version). Chinese Acupuncture & Moxibustion. 2008(11):854-9.

11. Li X, Liu X, Song W, Tang Y, Gao H, Zeng F, et al. Effect of Acupuncture on Cerebral Glucose Metabolism in Chronic Migraineurs: A PET-CT Study (Chinese Version). Journal of Chengdu University of TCM. 2008(03):1-5.

12. Ning Y, Zheng R, Lv Y, Fu C, Liu H, Ren Y. Study on the Influence of Acupuncture Zulinqi(GB41)on the Amplitude of Low Frequency Oscillation of Migraine (Chinese Version). World Chinese Medicine. 2020;15(20):3131-7.

13. Wu K, Xu L, Li K, Ren Y, Zou Y, Jiang L, et al. Effect of acupuncture on structual brain network in patients with different course of disease migraine without aura (Chinese Version). Journal of Traditional Chinese Medicine. 2020;61(24):2184-9.

14. Han X, Zou Y, Li K, Liu H, Ning Y, Tan Z, et al. Effect of acupunture at GB41 on migraine patients on the cortical regional homogeneity (ReHo) in the patient of chronic migraine (Chinese Version). Modern Chinese Clinical Medicine. 2017;24(06):31-5+65.

15. Liu H, Li K, Ning Y, Han X, Tan Z, Ren Y, et al. Effects of acupuncture at Zulinqi(GB41) on pain related brain networks of migraine patients: An fMRI study (Chinese Version). China Journal of Traditional Chinese Medicine and Pharmacy. 2016;31(05):2013-6.

16. Li K, Zhang Y, Ning Y, Zhang H, Liu H, Fu C, et al. The effects of acupuncture treatment on the right frontoparietal network in migraine without aura patients. J Headache Pain. 2015;16:518.

17. Liu J, Mu J, Chen T, Zhang M, Tian J. White matter tract microstructure of the mPFC-amygdala predicts interindividual differences in placebo response related to treatment in migraine patients. Hum Brain Mapp. 2019;40(1):284-92.

18. Liu J, Mu J, Liu Q, Dun W, Zhang M, Tian J. Brain structural properties predict psychologically mediated hypoalgesia in an 8-week sham acupuncture treatment for migraine. Hum Brain Mapp. 2017;38(9):4386-97.

19. Liu J, Ma S, Mu J, Chen T, Xu Q, Dun W, et al. Integration of white matter network is associated with interindividual differences in psychologically mediated placebo response in migraine patients. Hum Brain Mapp. 2017;38(10):5250-9.

20. Lin L, Ding R, Gu T. Effect of elecacupuncture on right thalamus and anterior cingulate gyrus Metabolism in Migraine: a magnetic resonance spectroscopy study (Chinese Version). Journal of Traditional Chinese Medicine. 2015;56(14):1220-3.

21. Lin L, Gu T, Ding R. Effect of acupuncture prophylaxis on left thalamus metabolism in migraine: a magnetic resonance spectroscopy study (Chinese Version). Chinese Journal of Clinical Healthcare. 2013;16(02):190-2.

22. Gu T, Lin L, Jiao S, Ding R. Effect of acupuncture prophylaxis on cerebral metabolism in migraine: a magnetic resonance spectroscopy study (Chinese Version). Journal of Medical Imaging. 2013;23(03):345-9.

23. Luo W, Zhang Y, Zhang Y, Zhou S, Yan Z, Liu B. Effect of auricular acupoint continuous stimulation on brain fraction amplitude of low-frequency fluctuation in patients with migraine without aura (Chinese Version). Chinese Imaging Journal of Integrated Traditional and Western Medicine. 2019;17(05):441-4.

24. Tan X, Wang W, Wang J, Xie W, Zhang Y, Gao Y. Analysis on regional homogeneity of resting brain during balance acupuncture-induced analgesiceffect in migraine patients without aura (Chinese Version). Acupuncture Research. 2019;44(06):446-50.

25. Zou Y, Tang W, Li X, Xu M, Li J. Acupuncture Reversible Effects on Altered Default Mode Network of Chronic Migraine Accompanied with Clinical Symptom Relief. Neural Plast. 2019;2019:5047463.

26. Zhang Y, Li KS, Liu HW, Fu CH, Chen S, Tan ZJ, et al. Acupuncture treatment modulates the resting-state functional connectivity of brain regions in migraine patients without aura. Chin J Integr Med. 2016;22(4):293-301.

27. Liang R, Zhang S, Xie Y. Study about Influence of Brain Metabolism in Patients with Chronic Migraine after Acupuncture at Shaoyang Specific Acupoints (Chinese Version). Chinese Archives of Traditional Chinese Medicine. 2016;34(04):918-20.

28. Chen X, Lin X, Xu X, Wu J. Effect of acupuncture at acupoints of the Shaoyang Meridian on diffusion tensor imaging in the patient of chronic migraine (Chinese Version). Chinese Journal of Integrative Medicine on Cardio-Cerebrovascular Disease. 2019;17(07):1092-3.
