# Supplementary material for: A Narrative Review of Neuroimaging Studies in Acupuncture for Migraine
Source: Pain Res Manag. 2021 Nov 10;2021:9460695. doi: 10.1155/2021/9460695 (PMC8598357; doi:10.1155/2021/9460695)
Supplement: Supplementary Materials — Supplementary Table 1. Full search strategy for each of the electronic databases queried. Supplementary Table 2. The basic information of the included studies. Supplementary Table 3. The study design of the included studies. Supplementary Table 4. The neuroimage information of the included studies. Supplementary Figure 1. The flow diagram of the literature search and screening process. Supplementary Figure 2. The basic information of the included studies. A. The annual distribution of included studies. B. The institution distribution of included studies. [file 9460695.f1.zip › Revised_Supplementary Table_4_scanning_information_reference.docx]

**Supplementary Table.4. The Neuroimage Information of the Included Studies.**

| Study  No. | Scanning Techniques | Analytical Methods | Scanning Results |
| --- | --- | --- | --- |
| S01(1) | fMRI | ALFF+ Machine Learning | After Acupuncture Treatment:  Bilateral MOG could effectively predict the relief of headache intensity of acupuncture treatment |
| S02(2) | fMRI | FC+ Machine Learning | After Acupuncture Treatment:  The neural marker - the visual, default mode, sensorimotor, and frontal-parietal networks for migraine without aura (MwoA) could response to real acupuncture. |
| S03(3) | fMRI | FC | VA Group:  ↑FC: Precuneus - MFG, Precuneus - Caudate |
| S04(4) | fMRI | ALFF/fALFF | VA Group:  ↑ALFF: RVM/TCC |
| S05(5) | fMRI | ICA+FC | After Acupuncture Treatment:  ↓FC: Precuneus - rFPN;  ↑FC: Precuneus - ACC , mPFC, Ventral Striatum, dPFC |
| S06(6) | fMRI | FC | After Acupuncture Treatment:  ↑FC: PAG-ACC |
| S07 (7) | fMRI | ReHo | ReHo (Active Acupoint Group > Inactive Acupoint Group): Thalamus, ACC, STC, SMA;  ReHo (Active Acupoint Group < Inactive Acupoint Group): Hippocampus, MFC, MTC |
| S08(8) | PET-CT | Calculate the Brain Glucose Metabolism | VA Group:  ↑Brain Glucose Metabolism: MFG, Postcentral Gyrus, precuneus, Parahippocampus, cerebellum and MCC;  ↓Brain Glucose Metabolism: MTC  SA Group:  ↑Brain Glucose Metabolism: PCC, Insula, ITC, MTC, STC, Postcentral Gyrus, Fusiform, IPL, SPL, Supramarginal gyrus, MOL, Angular gyrus, Precuneus  ↓Brain Glucose Metabolism: Cerebellum, Parahippocampus. |
| S09(9) | PET-CT | Calculate the Brain Glucose Metabolism | Traditional Group > Control Group: MTC, OFC, Insula, MFC, Angular Gyrus, PCC, Precuneus, MCC;  Traditional Group < Control Group: Parahippocampus, Hippocampus, Fusiform Gyrus, Postcentral Gyrus, Cerebellum |
| S10(10) | PET-CT | Calculate the Brain Glucose Metabolism | After Acupuncture Treatment:  ↓Brain Glucose Metabolism- Brainstem, Insula, Temporal Lobes |
| S11(11) | PET-CT | Calculate the Brain Glucose Metabolism | After Acupuncture Treatment:  ↓Brain Glucose Metabolism- Temporal Lobes |
| S12(12) | fMRI | ALFF/fALFF | Acupuncture Treatment for Migraine Patients:  ↑ALFF- Precentral gyrus, Postcentral gyrus;  ↓ALFF- Precuneus, MFC |
| S13(13) | DTI | White Matter Brain Network (TBSS-FA) | ↑Long course of diseases: the small world value, global network efficiency value of White matter network  ↓Short course of diseases: the small world value, global network efficiency value of White matter network |
| S14(14) | fMRI | ReHo | After Acupuncture Treatment:  ↓ReHo: MFC, SFC, tIFC |
| S15(15) | fMRI | Functional Brain Network (Functional Connectivity) | After Acupuncture Treatment:  ↑FC- Postcentral gyrus, Parahippocampus, Cingulate gyrus and Supramarginal gyrus |
| S16(16) | fMRI+DTI | FC | After Acupuncture Treatment:  ↑FC: Precentral Gyrus, Inferior Parietal Lobule, Postcentral Gyrus |
| S17(17) | DTI | TABS + Machine Learning | Placebo Acupuncture Treatment:  mPFC-Amygdala may be a predisposition for subsequent responses to placebo treatment |
| S18(18) | fMRI | VBM +FC+Machine Learning | Placebo Acupuncture Treatment:  Gray Matter Volume↓: mPFC  Functional connectivity↑: mPFC-DMN |
| S19(19) | DTI | White Matter Brain Networks (TBSS-FA) | Placebo Acupuncture Treatment:  ↑Within-Subnetwork Nodal Efficiency and Intersubnetwork Connectivity: Hippocampus-MFC |
| S20(20) | H-MRS | Calculate the H-MRS Metabolic Rations | Migraine Group>Headache Group:  ↑NAA /Cr ration at the Thalamus |
| S21(21) | H-MRS | Calculate the H-MRS Metabolic Rations | Migraine Group>Headache Group:  ↑NAA /Cr ration at the Thalamus |
| S22(22) | H-MRS | Calculate the H-MRS Metabolic Rations | Migraine Group>Headache Group:  ↑Cho/Cr: Thalamus  ↑NAA/Cr: Posterior Part of Paracentral Gyrus, Thalamus |
| S23(23) | fMRI | ALFF/fALFF | Vagus stimulation>Non-Vagus stimulation:  ↑fALFF: Solitary Nucleus, PCC, Thalamus, Cerebellum |
| S24(24) | fMRI | ReHo | Headache Acupoints Group:  ↑ReHo: ACC, Precentral gyrus, superior orbital frontal gyrus, Insula, inferior lobule, ACC, Ventral lateral nucleus and Ventral posteromedial nucleus of the thalamus, Pontine Nucleus, Cerebellar Tonsils and Orbital Frontal Inferior Gyrus;  ↓ReHo: ridge, Postcentral Gyrus, PCC, Precentral gyrus, Posterolateral Nucleus of Thalamus, and Hippocampus |
| S25(25) | fMRI | ICA | After Acupuncture Treatment:  ↑Functional connectivity - Superior Prefrontal Gyrus, Precuneus |
| S26(26) | fMRI | ICA | After Acupuncture Treatment:  ↑FC: Frontal and Temporal Lobe |
| S27(27) | H-MRS | Calculate the H-MRS Metabolic Rations | Treatment group＞Control group:  NAA /Cr ration at the PAG |
| S28(28) | DTI | TBSS | After Acupuncture Treatment:  ↓The Fractional Anisotropy of Hippocampus |

fMRI. functional Magnetic Resonance Imaging; MRI. Proton Magnetic Resonance Imaging; PET-CT. Positron Emission Tomography-computer tomography; H-MRS. Proton Magnetic Resonance Spectroscopy; DTI. Diffusion Tensor Imaging;

ALFF. Amplitude of Low Frequency Fluctuations; FC. Functional Connectivity; ReHo. Regional Homogeneity; ICA. Independent Components Analysis; TBSS. Tract Based Spatial Statistics

PAG. Periaqueductal Gray;

MFC. Middle Frontal Cortex; OFC. Orbital Frontal Cortex; SFC. Superior Frontal Cortex, tIFC. Triangular Part of Inferior Frontal Cortex

SMA. Supplementary Motor Area

mPFC. Medial Prefrontal Cortex; dPFC. Dorsolateral Prefrontal Cortex

ACC. Anterior Cingulate Cortex; MCC. Middle Cingulate Cortex; PCC. Posterior Cingulate Cortex;

MTC. Middle Temporal Cortex; IFC. Inferior Temporal Cortex; STC. Superior Temporal Cortex

IPL. Inferior Parietal Lobe; SPL. Superior Parietal Lobe

MOL. Middle Occipital Lobe; MOG. Middle Occipital Gyrus

RVM. Rostral Ventromedial Medulla; TCC, Trigeminocervical Complex

DMN. Default Mode Network; FPN. Frontoparietal Network

**Reference**

1. Yin T, Sun G, Tian Z, Liu M, Gao Y, Dong M, et al. The Spontaneous Activity Pattern of the Middle Occipital Gyrus Predicts the Clinical Efficacy of Acupuncture Treatment for Migraine Without Aura. Front Neurol. 2020;11:588207.

2. Tu Y, Zeng F, Lan L, Li Z, Maleki N, Liu B, et al. An fMRI-based neural marker for migraine without aura. Neurology. 2020;94(7):e741-e51.

3. Zhang Y, Xu T, Wang X, Wang Z, Du J, Zhao L. Exploration on the effects of acupuncture on the precuneus functional connectivity of menstrual migraine patients by fMRI (Chinese Version). China Journal of Traditional Chinese Medicine and Pharmacy. 2020;35(02):1002-6.

4. Li Z, Zeng F, Yin T, Lan L, Makris N, Jorgenson K, et al. Acupuncture modulates the abnormal brainstem activity in migraine without aura patients. Neuroimage Clin. 2017;15:367-75.

5. Li Z, Lan L, Zeng F, Makris N, Hwang J, Guo T, et al. The altered right frontoparietal network functional connectivity in migraine and the modulation effect of treatment. Cephalalgia. 2017;37(2):161-76.

6. Li Z, Liu M, Lan L, Zeng F, Makris N, Liang Y, et al. Altered periaqueductal gray resting state functional connectivity in migraine and the modulation effect of treatment. Sci Rep. 2016;6:20298.

7. Zhao L, Liu J, Zhang F, Dong X, Peng Y, Qin W, et al. Effects of long-term acupuncture treatment on resting-state brain activity in migraine patients: a randomized controlled trial on active acupoints and inactive acupoints. PLoS One. 2014;9(6):e99538.

8. Yang M, Yang J, Zeng F, Liu P, Lai Z, Deng S, et al. Electroacupuncture stimulation at sub-specific acupoint and non-acupoint induced distinct brain glucose metabolism change in migraineurs: a PET-CT study. J Transl Med. 2014;12:351.

9. Yang J, Zeng F, Feng Y, Fang L, Qin W, Liu X, et al. A PET-CT study on the specificity of acupoints through acupuncture treatment in migraine patients. BMC Complement Altern Med. 2012;12:123.

10. Li X, Liu X, Song W, Tang Y, Zeng F, Liang F. Effect of acupuncture at acupoints of the Shaoyang Meridian on cerebral glucose metabolism in the patient of chronic migraine (Chinese Version). Chinese Acupuncture & Moxibustion. 2008(11):854-9.

11. Li X, Liu X, Song W, Tang Y, Gao H, Zeng F, et al. Effect of Acupuncture on Cerebral Glucose Metabolism in Chronic Migraineurs: A PET-CT Study (Chinese Version). Journal of Chengdu University of TCM. 2008(03):1-5.

12. Ning Y, Zheng R, Lv Y, Fu C, Liu H, Ren Y. Study on the Influence of Acupuncture Zulinqi(GB41)on the Amplitude of Low Frequency Oscillation of Migraine (Chinese Version). World Chinese Medicine. 2020;15(20):3131-7.

13. Wu K, Xu L, Li K, Ren Y, Zou Y, Jiang L, et al. Effect of acupuncture on structual brain network in patients with different course of disease migraine without aura (Chinese Version). Journal of Traditional Chinese Medicine. 2020;61(24):2184-9.

14. Han X, Zou Y, Li K, Liu H, Ning Y, Tan Z, et al. Effect of acupunture at GB41 on migraine patients on the cortical regional homogeneity (ReHo) in the patient of chronic migraine (Chinese Version). Modern Chinese Clinical Medicine. 2017;24(06):31-5+65.

15. Liu H, Li K, Ning Y, Han X, Tan Z, Ren Y, et al. Effects of acupuncture at Zulinqi(GB41) on pain related brain networks of migraine patients: An fMRI study (Chinese Version). China Journal of Traditional Chinese Medicine and Pharmacy. 2016;31(05):2013-6.

16. Li K, Zhang Y, Ning Y, Zhang H, Liu H, Fu C, et al. The effects of acupuncture treatment on the right frontoparietal network in migraine without aura patients. J Headache Pain. 2015;16:518.

17. Liu J, Mu J, Chen T, Zhang M, Tian J. White matter tract microstructure of the mPFC-amygdala predicts interindividual differences in placebo response related to treatment in migraine patients. Hum Brain Mapp. 2019;40(1):284-92.

18. Liu J, Mu J, Liu Q, Dun W, Zhang M, Tian J. Brain structural properties predict psychologically mediated hypoalgesia in an 8-week sham acupuncture treatment for migraine. Hum Brain Mapp. 2017;38(9):4386-97.

19. Liu J, Ma S, Mu J, Chen T, Xu Q, Dun W, et al. Integration of white matter network is associated with interindividual differences in psychologically mediated placebo response in migraine patients. Hum Brain Mapp. 2017;38(10):5250-9.

20. Lin L, Ding R, Gu T. Effect of elecacupuncture on right thalamus and anterior cingulate gyrus Metabolism in Migraine: a magnetic resonance spectroscopy study (Chinese Version). Journal of Traditional Chinese Medicine. 2015;56(14):1220-3.

21. Lin L, Gu T, Ding R. Effect of acupuncture prophylaxis on left thalamus metabolism in migraine: a magnetic resonance spectroscopy study (Chinese Version). Chinese Journal of Clinical Healthcare. 2013;16(02):190-2.

22. Gu T, Lin L, Jiao S, Ding R. Effect of acupuncture prophylaxis on cerebral metabolism in migraine: a magnetic resonance spectroscopy study (Chinese Version). Journal of Medical Imaging. 2013;23(03):345-9.

23. Luo W, Zhang Y, Zhang Y, Zhou S, Yan Z, Liu B. Effect of auricular acupoint continuous stimulation on brain fraction amplitude of low-frequency fluctuation in patients with migraine without aura (Chinese Version). Chinese Imaging Journal of Integrated Traditional and Western Medicine. 2019;17(05):441-4.

24. Tan X, Wang W, Wang J, Xie W, Zhang Y, Gao Y. Analysis on regional homogeneity of resting brain during balance acupuncture-induced analgesiceffect in migraine patients without aura (Chinese Version). Acupuncture Research. 2019;44(06):446-50.

25. Zou Y, Tang W, Li X, Xu M, Li J. Acupuncture Reversible Effects on Altered Default Mode Network of Chronic Migraine Accompanied with Clinical Symptom Relief. Neural Plast. 2019;2019:5047463.

26. Zhang Y, Li KS, Liu HW, Fu CH, Chen S, Tan ZJ, et al. Acupuncture treatment modulates the resting-state functional connectivity of brain regions in migraine patients without aura. Chin J Integr Med. 2016;22(4):293-301.

27. Liang R, Zhang S, Xie Y. Study about Influence of Brain Metabolism in Patients with Chronic Migraine after Acupuncture at Shaoyang Specific Acupoints (Chinese Version). Chinese Archives of Traditional Chinese Medicine. 2016;34(04):918-20.

28. Chen X, Lin X, Xu X, Wu J. Effect of acupuncture at acupoints of the Shaoyang Meridian on diffusion tensor imaging in the patient of chronic migraine (Chinese Version). Chinese Journal of Integrative Medicine on Cardio-Cerebrovascular Disease. 2019;17(07):1092-3.
