# Supplementary material for: A Narrative Review of Neuroimaging Studies in Acupuncture for Migraine
Source: Pain Res Manag. 2021 Nov 10;2021:9460695. doi: 10.1155/2021/9460695 (PMC8598357; doi:10.1155/2021/9460695)
Supplement: Supplementary Materials — Supplementary Table 1. Full search strategy for each of the electronic databases queried. Supplementary Table 2. The basic information of the included studies. Supplementary Table 3. The study design of the included studies. Supplementary Table 4. The neuroimage information of the included studies. Supplementary Figure 1. The flow diagram of the literature search and screening process. Supplementary Figure 2. The basic information of the included studies. A. The annual distribution of included studies. B. The institution distribution of included studies. [file 9460695.f1.zip › Revised_Supplementary_Figure1.pptx]

## Slide 1
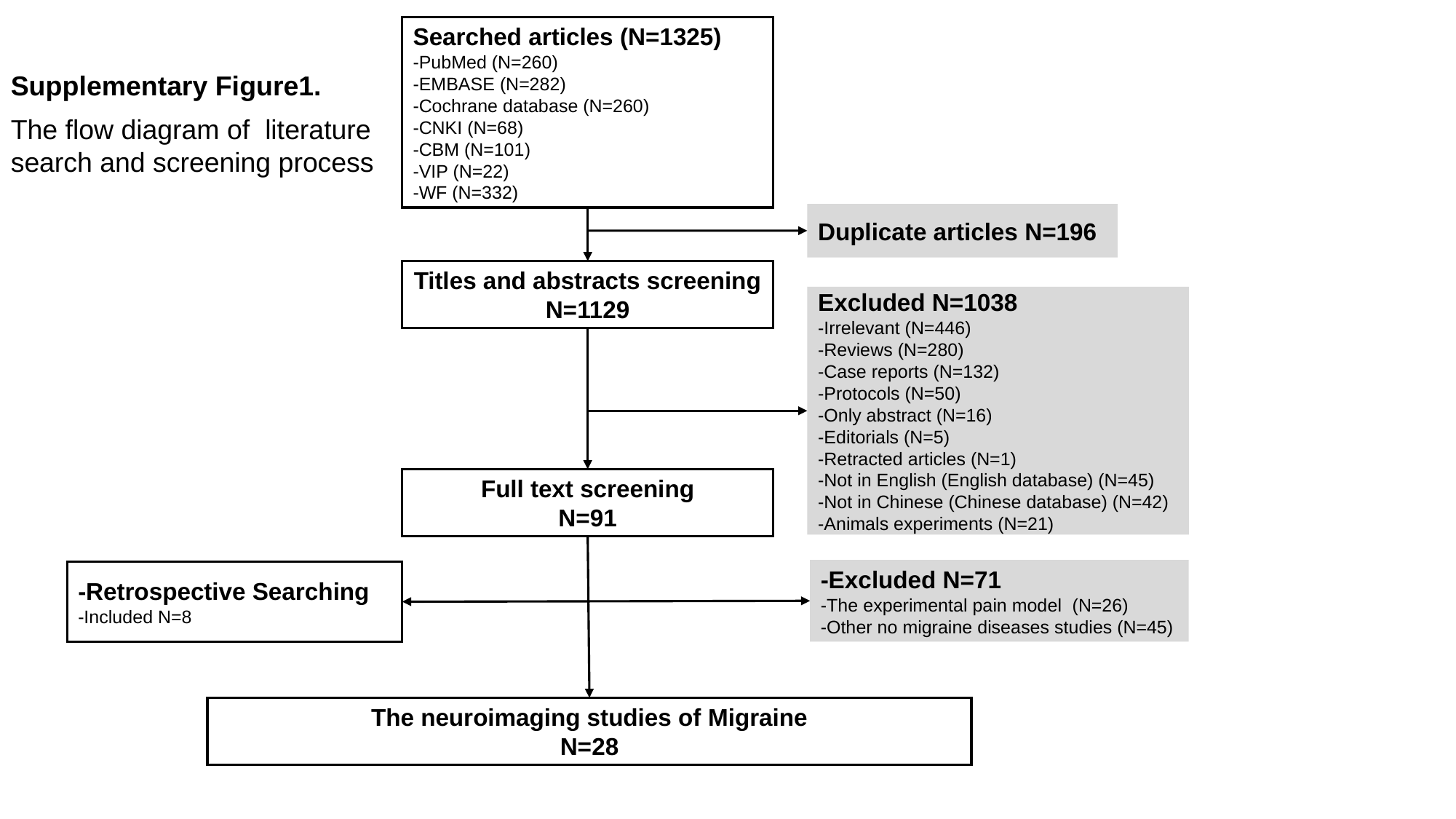

Searched articles (N=1325)
-PubMed (N=260)
-EMBASE (N=282)
-Cochrane database (N=260)
-CNKI (N=68)
-CBM (N=101)
-VIP (N=22)
-WF (N=332)
Supplementary Figure1.
The flow diagram of literature search and screening process
Duplicate articles N=196
Titles and abstracts screening
N=1129
Excluded N=1038
-Irrelevant (N=446)
-Reviews (N=280)
-Case reports (N=132)
-Protocols (N=50)
-Only abstract (N=16)
-Editorials (N=5)
-Retracted articles (N=1)
-Not in English (English database) (N=45)
-Not in Chinese (Chinese database) (N=42)
-Animals experiments (N=21)
Full text screening
N=91
-Excluded N=71
-The experimental pain model (N=26)
-Other no migraine diseases studies (N=45)
-Retrospective Searching
-Included N=8
The neuroimaging studies of Migraine
N=28
