# Supplementary material for: A Narrative Review of Neuroimaging Studies in Acupuncture for Migraine
Source: Pain Res Manag. 2021 Nov 10;2021:9460695. doi: 10.1155/2021/9460695 (PMC8598357; doi:10.1155/2021/9460695)
Supplement: Supplementary Materials — Supplementary Table 1. Full search strategy for each of the electronic databases queried. Supplementary Table 2. The basic information of the included studies. Supplementary Table 3. The study design of the included studies. Supplementary Table 4. The neuroimage information of the included studies. Supplementary Figure 1. The flow diagram of the literature search and screening process. Supplementary Figure 2. The basic information of the included studies. A. The annual distribution of included studies. B. The institution distribution of included studies. [file 9460695.f1.zip › Revised_Supplementary_Figure2.pptx]

## Slide 1
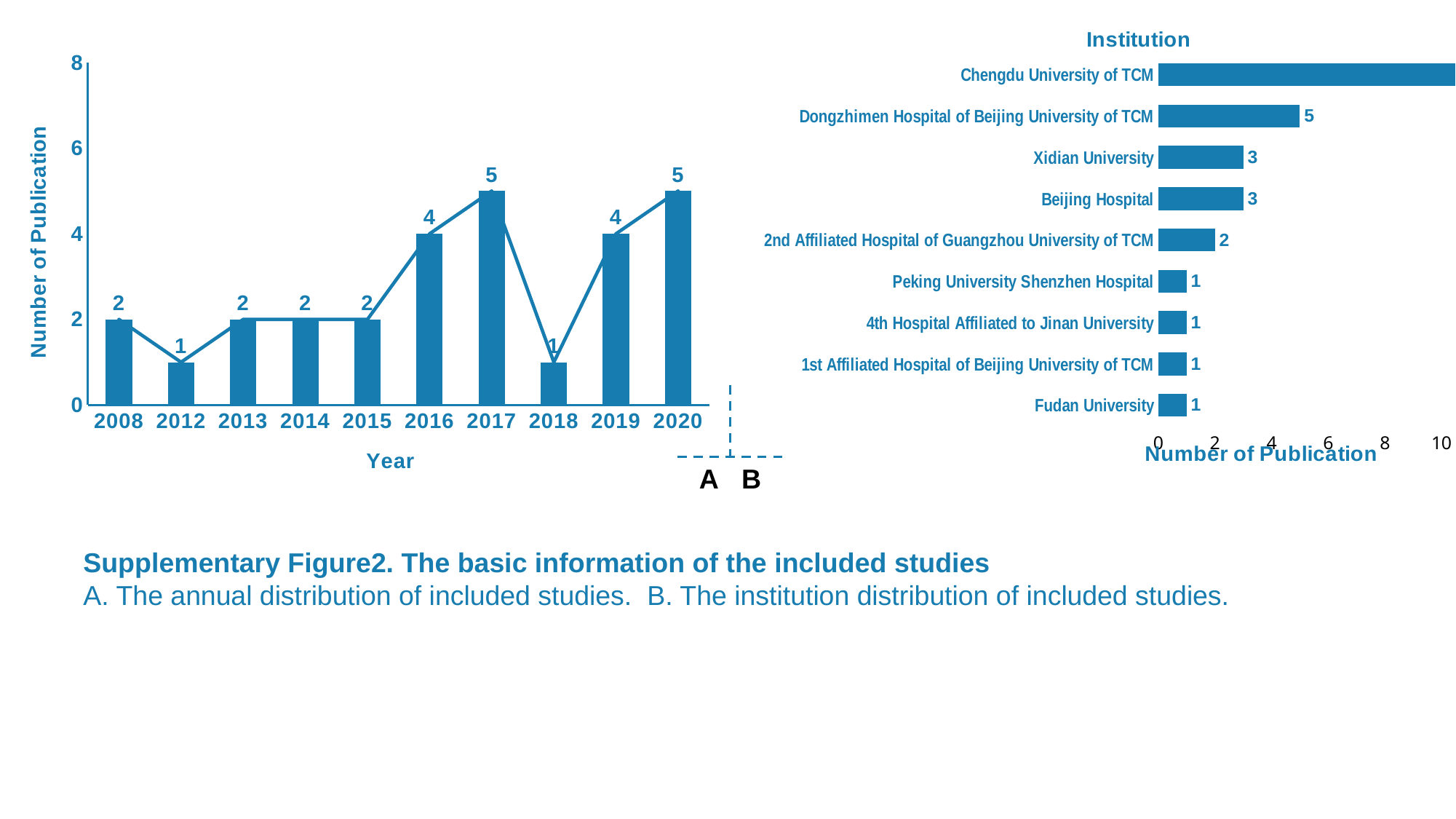

### Chart: Institution
| Category | Instuition |
|---|---|
| Fudan University | 1.0 |
| 1st Affiliated Hospital of Beijing University of TCM | 1.0 |
| 4th Hospital Affiliated to Jinan University | 1.0 |
| Peking University Shenzhen Hospital | 1.0 |
| 2nd Affiliated Hospital of Guangzhou University of TCM | 2.0 |
| Beijing Hospital | 3.0 |
| Xidian University | 3.0 |
| Dongzhimen Hospital of Beijing University of TCM | 5.0 |
| Chengdu University of TCM | 11.0 |
### Chart
| Category | 年份 | 年份2 |
|---|---|---|
| 2008 | 2.0 | 2.0 |
| 2012 | 1.0 | 1.0 |
| 2013 | 2.0 | 2.0 |
| 2014 | 2.0 | 2.0 |
| 2015 | 2.0 | 2.0 |
| 2016 | 4.0 | 4.0 |
| 2017 | 5.0 | 5.0 |
| 2018 | 1.0 | 1.0 |
| 2019 | 4.0 | 4.0 |
| 2020 | 5.0 | 5.0 |A
B
Supplementary Figure2. The basic information of the included studies
A. The annual distribution of included studies. B. The institution distribution of included studies.
